# Exploring the association of time-inconsistent preferences with smoking behavior: A cross-sectional survey study from Sichuan, China

**DOI:** 10.18332/tid/209192

**Published:** 2025-09-24

**Authors:** Yanru Li, Shiyao Ling, Yuelin Zhou, Yong Li, Jingman He, Lian Yang

**Affiliations:** 1 School of Management, Chengdu University of Traditional Chinese Medicine, Chengdu, China; 2 School of Public Health, Chengdu University of Traditional Chinese Medicine, Chengdu, China

**Keywords:** time-inconsistent preference, smoking behavior, tobacco and alcohol co-use, quasi-hyperbolic discounting model

## Abstract

**INTRODUCTION:**

The high prevalence of tobacco use in China has led to a heavy social burden. There have been many studies on smoking behavior in China, but few have explored it from the perspective of behavioral economics. This study investigated the association between time-inconsistent preferences and smoking behavior.

**METHODS:**

We conducted a household-based cross-sectional survey in Sichuan Province, southwestern China, in 2022. Participants were selected using multistage stratified sampling. Data were collected through face-to-face, questionnaire-based household surveys, yielding 5446 valid responses. The smoking status of all participants was confirmed by cotinine test. Descriptive statistical analysis was performed on variables such as sociodemographic variables and time-inconsistent preference, and a binary logistic regression model was used to explore the relationship between time-inconsistent preference and smoking behavior.

**RESULTS:**

The smoking rate of the survey participants was 23.87%, among which that of drinkers was 45.90%, which was significantly higher than that of non-drinkers (15.10%); 712 participants (13.07%) both smoked and drank, and the average time-inconsistent preference (time-inconsistent preferences measured over 1-month and 12-month delay periods) of smokers (mean ± standard deviation: 0.91 ± 0.14) was significantly lower than that of non-smokers (0.95 ± 0.11, p<0.001). Thus, those with weaker time-inconsistent preferences were less likely to smoke (p<0.001, adjusted odds ratio, AOR=0.276; 95% CI: 0.140–0.544). This trend was significant in both drinkers and non-drinkers, with AOR of 0.253 (95% CI: 0.093–0.693) and 0.26 (95% CI: 0.102–0.661), respectively.

**CONCLUSIONS:**

Weaker time-inconsistent preferences show a significant negative association with smoking behavior in this Chinese sample, independent of alcohol consumption. Commitment mechanisms targeting time-inconsistent preferences may hold promise for smoking cessation interventions, although efficacy requires validation through randomized trials.

## INTRODUCTION

The tobacco epidemic is one of the greatest public health threats the world has ever faced, causing more than 8 million deaths worldwide each year, of which more than 7 million are directly caused by tobacco use^1^. Of the 1.3 billion tobacco users in the world, about 80% live in low- and middle-income countries, including China, where the burden of tobacco-related disease and death is the highest^2^. China is currently the world’s largest producer, distributor, and consumer of tobacco. The country consumes 40% of the world’s tobacco, with more than 1 million Chinese people dying each year from various diseases caused by smoking, and this number continues to rise^3^.

With the rise of the behavioral economics field, represented by Daniel Kahneman, smoking is now considered an intertemporal decision-making behavior^4^ – smokers need to choose between the ‘immediate pleasure’ and the future ‘health damage’ brought by smoking. When making intertemporal decisions, people experience projection bias and hindsight bias, with the former referring to predictions of how they might feel at certain future moments and the latter referring to retrospection on the information available at the time a judgment was made in the past^5^. These biases give rise to time-inconsistent preferences, which refer to individuals’ beliefs at certain points in time that they will have a set of preferences in the future, but their previous expectations turn out to be incorrect, and this inconsistency triggers ambivalence and, in turn, induces feelings of regret. The development of addictive habits, such as smoking and excessive drinking, is one of the typical manifestations of such regret^6^. Several studies have shown that time-inconsistent preferences are associated with unhealthy behaviors^7^. A large number of studies have shown that time-inconsistent preferences are associated with smoking behavior^8^. When making smoking-related decisions, smokers often believe that the current happiness brought by smoking is greater than the future benefits of quitting smoking. Therefore, they choose not to quit smoking now but to put it off until later. When the day to quit comes, they again choose to continue smoking^9^. Moreover, the more severe the time-inconsistent preference is, the more likely it is to be associated with smoking^10^. Although many foreign studies have shown that time-inconsistent preferences may be an important factor linked to smoking behavior, in China there is still a lack of research on the association between the two. This study aims to analyze the association of time-inconsistent preferences with smoking behavior to fill this gap.

Alcohol drinkers exhibit tendencies similar to those of smokers. Studies have shown that individuals who show higher time discount rates in intertemporal decision-making (i.e. the more severe the degree of time-inconsistent preference) are more likely to drink and that drinkers are more likely to choose immediate gratification than potential future benefits^11^. As Shiffman and Balabanis^12^, ‘drinkers smoke and smokers drink’, indicating the close association between smoking and drinking behavior^13,14^. Alcohol is deeply rooted in traditional Chinese culture, with tobacco and alcohol often being used as ‘lubricants’ in social situations. This leads to a high prevalence of combined tobacco and alcohol use^15^. Studies have also shown that drinking increases the desire to smoke^16^ and the occurrence of smoking behavior^17^. Many studies have found that Chinese drinkers have significantly higher smoking rates than non-drinkers^18,19^. Given that drinking behavior is closely related to smoking behavior, does drinking behavior, as a confounding factor, affect the association between time-inconsistent preference and smoking behavior? Is time-inconsistent preference associated with smoking behavior independently of drinking behavior? To gain a deeper understanding of this complex relationship, it is necessary to analyze and discuss drinkers and non-drinkers separately.

Most current studies on intertemporal choice behavior employ the quasi-hyperbolic discounting model^20^. Compared with the former hyperbolic discounting model^21^, the quasi-hyperbolic discounting model is simpler to solve, and Laibson^22^ added a discount factor on the basis of the hyperbolic discounting function, which effectively simulates the inconsistency of consumers’ time preferences and greatly broadens the application field of hyperbolic discounting. Therefore, this study employs the quasi-hyperbolic model to measure time-inconsistent preferences and examine its relationship with smoking behavior. Further, stratified analyses are conducted according to the drinking habits of the participants to exclude the potential confounding factor of alcohol consumption and validate the association between time-inconsistent preferences with smoking behavior.

## METHODS

### Participants and procedures

This study employed a cross-sectional design, utilizing literature review and questionnaire-based data collection. Data were gathered from January 2022 to April 2023. Using multistage stratified cluster random sampling, individuals aged ≥15 years were selected from four regions in Sichuan Province: Wenjiang District (Chengdu), Fushun County (Zigong), Qingchuan County (Guangyuan), and Xide County (Liangshan Prefecture). Trained interviewers administered structured questionnaires during household visits. Participants were screened based on the following criteria:

Inclusion criteria: 1) age ≥15 years; 2) permanent residency in the surveyed districts/counties for ≥6 months; and 3) normal communication ability. Exclusion criteria were: 1) impaired communication capacity (e.g. severe illness or cognitive deficiency); and 2) explicit refusal to participate.

All interviewers received standardized professional training to ensure data reliability. After collection, rigorous data cleaning excluded entries with logical errors or inaccuracies. Smoking status was biochemically verified using salivary cotinine testing. Participants were classified as smokers only if both self-reported smoking and cotinine test results were positive; otherwise, they were categorized as non-smokers.

A cotinine rapid test kit (sensitivity: 30 ppb) measured salivary cotinine concentration. The on-site procedure followed the manufacturer’s protocol strictly:

1. The participants rinsed their mouths with water.
2. The interviewer collected approximately 1 mL of saliva using a collection cup.
3. Using a dropper, 2–3 drops of saliva were transferred to the test strip sample well.
4. Results were read after a 5-minute incubation period.

The study protocol received approval from the Medical Ethics Committee of the Affiliated Hospital of Chengdu University of Traditional Chinese Medicine (Approval No. 2023KL-134).

### Measurements

*Measurement of self-control*

The participants’ self-control was measured using the Self-Control Scale, which was published by Tangney in 2004 and has been widely used in the measurement of self-control since being revised by Tan and Guo^23^. The scale has 19 items in total, using the 5-point Likert rating system. Each item is scored depending on the level of consistency (i.e. completely inconsistent, somewhat inconsistent, uncertain, relatively consistent and completely consistent). The scale contains five dimensions, namely impulse control, healthy habits, resisting temptation, focusing on work, and abstaining from entertainment. A higher total score indicates better self-control. In this study, the reliability analysis of the scale yielded a Cronbach’s α coefficient of 0.824, indicating good reliability.

*Measurement of emotion*

Emotion was measured using the Chinese version of the Positive and Negative Affect Schedule (PANAS)^24^. The scale consists of positive affect (PA) and negative affect (NA) subscales, containing 10 adjectives describing positive emotions (interested, energetic, determined, etc.) and negative emotions (upset, guilty, fearful, etc.), respectively. Each question is rated for intensity on a 5-point Likert scale (i.e. from almost no intensity to relatively little intensity, moderate intensity, relatively intense, and very intense). Those with higher NA scores suffer from more negative emotions. Since positive and negative emotions are two different and independent dimensional characteristics, this study only used the NA subscale. In this study, the retest reliability of the NA subscale was good, with a Cronbach’s α coefficient of 0.876.

*Measurement of time-inconsistent preference*

Time-inconsistent preferences were measured based on participants’ intertemporal decision-making behavior. MEL (Money Earlier or Later) is the most common method used in the laboratory to examine time-inconsistent preferences. This method is divided into three categories: matching (or fill-in-the-blank), multiple price, and random binary choice design^25,26^. This study used the multiple price design method to design the questionnaire. That is, the participants had to choose between obtaining an amount at an earlier date and obtaining an amount at a later date, with the former always being less than the latter. The earlier and later dates were fixed, as was the amount obtained at the earlier date, and the amount set for the later date changed monotonically so that the difference between the amounts on the two dates gradually widened. When conducting a multiple price design experiment, the participants are usually told that after making all their choices, a decision will be randomly selected, and a betting game will be played according to the selection, and finally, the reward will be obtained according to the game’s result. To ensure that the questionnaire truly reflected their situation, the participants in this study were informed before measuring their intertemporal decision-making behavior that they would be rewarded by rolling dice after performing their intertemporal decision-making behavior. The surveyor paid them cash according to the time and amount selected in the question^27^ (See the Supplementary file for specific selection scenarios). Laibson’s quasi-hyperbolic discounting model was then used to measure time-inconsistent preference^28^:


$$\text{U}\left(\text{t},\text{s}\right)={\text{u}}_{\text{t}}+\text{β}\sum_{\text{s}=\text{t}+1}^{\text{∞}}{\text{δ}}^{\text{s}-1}\;{\text{u}}_{\text{s}}$$

where β is the short-term discount factor, which represents individual time-inconsistent preference and is used to study the problem of self-restraint. When β<1, it means that in any given period, the individual prefers to receive a benefit in the present rather than in the future; δ is the long-term discount factor, and β×δ is the discount factor between the current decision period and the next period. In general, β and δ are between 0 and 1. At this time, β×δ < δ, indicating that the short-term discount factor is smaller than the long-term discount factor, reflecting the characteristic of decreasing individual impatience. When β=1, it means that the short-term discount factor is equal to the long-term discount factor, and the individual's time-inconsistent preference is more serious. This study used the short-term discount factor β to reflect the individual's time-inconsistent preference.

*Measurement of sociodemographic variables*

Sociodemographic data were collected via structured questionnaires. Variables included: age was measured as a continuous variable (years) and categorized into three groups: 15–44 years, 45–59 years, and ≥60 years. Gender was recorded as a binary variable (male, female), as was household registration (agricultural, non-agricultural). Ethnicity was classified as Han or Other. Education level was categorized as: No schooling, Primary school, Secondary school (including junior high, senior high, technical school, or secondary specialized school), or Higher education (including college, undergraduate, or postgraduate). Marital status was categorized as unmarried, married, or other (divorced/widowed). Income was assessed as a binary variable relative to the 2023 Sichuan provincial per capita disposable income, categorized as: ≤ average income or > average income. Employment status was recorded as employed or unemployed. Alcohol use was defined based on the question: ‘Have you consumed any alcoholic beverage in the past 12 months?’, with respondents classified as non-drinkers (‘No’) or drinkers (‘Yes’).

### Variable specification

The primary outcome variable was biochemically verified smoking status (smoker vs non-smoker). The key predictor variable was time-inconsistent preference (β). Covariates treated as potential confounders included: Behavioral factor: alcohol use (drinker, non-drinker); sociodemographic factors: age, gender, household registration, ethnicity, education level, marital status, income, and employment status; Psychological factors: self-control and negative emotion.

### Statistical analysis

SPSS 22.0 was used for statistical analysis. Descriptive statistics were performed on sociodemographic variables (age, gender, marital status, education level, income, household registration type, employment status, ethnicity, and use of alcohol), time-inconsistent preference, self-control, and negative emotions. Categorical variables are expressed as frequencies and percentages, and continuous variables as mean ± standard deviation (SD). Categorical variables are expressed as frequencies and percentages, and the chi-squared test was used to detect significant differences between them and smoking behavior. Continuous variables were expressed as mean ± standard deviation (SD) and normality was tested by the Shapiro-Wilk test. Normal variables were analyzed using the independent sample t-test, and non-normal variables were analyzed using the Mann-Whitney U test. Multivariable binary logistic regression analyzed factors associated with smoking behavior. Stratified binary logistic regression by alcohol use (drinker, non-drinker) examined the association between time-inconsistent preferences and smoking while controlling for potential confounding by alcohol. Statistical significance was set at p<0.05. In all models, time-inconsistent preferences (β) was the primary exposure variable, and biochemically verified smoking status was the outcome. Stratification by alcohol use aimed to control its potential confounding effect on the time-inconsistent preferences-smoking association.

## RESULTS

### Participant demographics

Participants (n=5446) had a mean age of 47.08 years; 45.0% (n=2450) were male. The majority held agricultural household registration (64.7%, n=3525) and identified as Han ethnicity (90.4%, n=4924). Of the cohort, 1300 (23.87%) were smokers and 4146 (76.13%) were non-smokers. Smoking prevalence was significantly higher among drinkers (45.90%) compared to non-drinkers (15.10%), and 712 participants (13.07%) reported both smoking and drinking. The average time-inconsistent preference score of smokers was 0.91 ± 0.14, which was lower than that of non-smokers (0.95 ± 0.11). There were significant differences between smokers and non-smokers in gender, use of alcohol, household registration type, ethnicity, education level, marital status, income, employment status, time-inconsistent preference, self-control, and negative emotions (all p<0.05) (Table 1).

**Table 1:** Characteristics of study population, Sichuan Province, China, 2022 (N=5446)

*Characteristics*	*Categories*	*Total*	*Non-smokers*		*Smokers*		*χ^2^*	*p*
		*n*	*n*	*%*	*n*	*%*		
**Gender**	Male	2450	1235	50.40	1215	49.60	1621.302	<0.001
	Female	2996	2911	97.20	85	2.80		
**Alcohol use**	Non-drinker	3896	3308	84.90	588	15.10	580.458	<0.001
	Drinker	1550	838	54.10	712	45.90		
**Age** (years)	15–44	2315	1776	76.70	539	23.30	2.257	0.324
	45–59	1520	1136	74.70	384	25.30		
	≥60	1611	1234	76.60	377	23.40		
**Household registration type**	Agricultural	3525	2613	74.10	912	25.90	22.032	<0.001
	Non-agricultural	1921	1533	79.80	388	20.20		
**Ethnicity**	Han	4924	3805	77.30	1119	22.70	37.081	<0.001
	Other	522	341	65.30	181	34.70		
**Education level**	No schooling	670	550	82.10	120	17.90		
	Primary school	1346	994	73.80	352	26.20	48.985	<0.001
	Secondary school	2289	1670	73.00	619	27.00		
	Higher education	1141	932	81.70	209	18.30		
**Marital status**	Unmarried	1263	981	77.70	282	22.30	11.812	0.003
	Married	3793	2845	75.00	948	25.00		
	Other	390	320	82.10	70	17.90		
**Income^a^**	≤ average	3652	2909	79.70	743	20.30	75.834	<0.001
	> average	1794	1237	69.00	557	31.00		
**Employment**	Employed	2511	1707	68.00	804	32.00	170.232	<0.001
	Unemployed	2935	2439	83.10	496	16.90		
		***Mean ± SD***	***Mean ± SD***		***Mean ± SD***		***Z***	***p***
**Time-inconsistent preference^b^**		0.94 ± 0.12	0.95 ± 0.11		0.91 ± 0.14		-9.702	<0.001
**Self-control^c^**		68.63 ± 9.26	69.61 ± 8.92		65.53 ± 9.64		-13.033	<0.001
**Negative emotions^d^**		17.52 ± 6.22	17.69 ± 6.26		16.96 ± 6.03		-3.803	<0.001

a Average income based on Sichuan provincial per capita disposable income.

b Time-inconsistent preference measured by quasi-hyperbolic discounting model (β), range 0–1 (higher=stronger preference for immediate rewards).

c Self-control using Tangney’s Self-Control Scale (19 items, Likert 1–5), total score 19–95.

d Negative emotions using PANAS negative affect subscale (10 items, Likert 1–5), total score 10–50.

Further analysis of the factors associated with smoking behavior revealed that gender (male reference: AOR=0.035; 95% CI: 0.027–0.044, p<0.001), alcohol use (non-drinker reference: AOR=2.266; 95% CI: 1.915–2.68, p<0.001), ethnicity (Han reference: AOR=1.707; 95% CI: 1.298–2.246, p<0.001), education level (no schooling reference: primary school, AOR=1.016; 95% CI: 0.735–1.403; secondary school, AOR=1.034; 95% CI: 0.746–1.433; higher education, AOR=1.707; 95% CI: 1.298–2.246, p<0.01), marital status (unmarried reference: married, AOR=1.454; 95% CI: 1.121–1.887; other, AOR=1.729; 95% CI: 1.117–2.674; p=0.01), employment status (employed reference: AOR=0.507; 95% CI: 0.417–0.618, p<0.001), time-inconsistent preference (AOR=0.276; 95% CI: 0.140–0.544, p<0.001), self-control (p<0.001, AOR=0.939; 95% CI: 0.929–0.948), and negative emotions (p=0.01, AOR=0.975; 95% CI: 0.961–0.99) were significantly associated with smoking behavior. It also revealed that those with weaker time-inconsistent preferences were less likely to smoke (p<0.001, AOR=0.276; 95% CI: 0.140–0.544) .For each 0.01-unit increase in the time-inconsistent preference factor (β, where higher β indicates weaker preference for immediate gratification), the likelihood of smoking decreased (AOR=0.276 per 1-unit increase in β, 95% CI: 0.140–0.544; p<0.001).Drinkers were more likely to smoke than non-drinkers (AOR=2.266; 95% CI: 1.915–2.680; p<0.001) (Table 2).

**Table 2:** Multivariable logistic regression for smoking behavior, Sichuan Province, China, 2022 (N=5446)

*Variables*	*AOR*	*95% CI*		*p*
		*Lower*	*Upper*	
**Gender**				<0.001
Male ®	1			
Female	0.035	0.027	0.044	
**Alcohol use**				<0.001
Non-Drinker ®	1			
Drinker	2.266	1.915	2.68	
**Age** (years)				0.874
15–44 ®	1			
45–59	1.066	0.828	1.372	
≥60	1.064	0.8	1.415	
**Household registration type**				0.074
Agricultural ®	1			
Non-agricultural	0.843	0.699	1.017	
**Ethnicity**				<0.001
Han ®	1			
Other	1.707	1.298	2.246	
**Education level**				<0.001
No schooling ®	1			
Primary school	1.016	0.735	1.403	
Secondary school	1.034	0.746	1.433	
Higher education	0.539	0.366	0.794	
**Marital status**				0.01
Unmarried ®	1			
Married	1.454	1.121	1.887	
Other	1.729	1.117	2.674	
**Income**				0.298
≤ average ®	1			
> average	1.109	0.913	1.348	
**Employment**				<0.001
Employed ®	1			<0.001
Unemployed	0.507	0.417	0.618	
**Time-inconsistent preference**	0.276	0.14	0.544	
**Self-control**	0.939	0.929	0.948	<0.001
**Negative emotions**	0.975	0.961	0.99	0.001

AOR: adjusted odds ratio; adjusted for all sociodemographic variables, self-control, and negative emotions. ® Reference categories.

**Table 3:** Characteristics of smoking among non-drinkers and drinkers, Sichuan Province, China, 2022 (N=5446)

*Variables*	*Non-drinkers*						*Drinkers*					
	*Non-smokers*		*Smokers*		*Parametric/* *non-parametric test*		*Non-smokers*		*Smokers*		*Parametric/* *non-parametric test*	
	*n*	*%*	*n*	*%*	*χ^2^*	*p*	*n*	*%*	*n*	*%*	*χ^2^*	*p*
**Gender**					1024.70	<0.001**^a^**					340.01	<0.001**^a^**
Male	798	59.60	542	40.40			437	39.40	673	60.60		
Female	2510	98.20	46	1.80			401	91.10	39	8.90		
**Age** (years)					0.42	0.809**^a^**					10.54	0.005**^a^**
15–44	1363	85.20	236	14.80			413	57.70	303	42.30		
45–59	936	85.00	165	15.00			200	47.70	219	52.30		
≥60	1009	84.40	187	15.60			225	54.20	190	45.80		
**Household registration type**					17.26	<0.001**^a^**					12.12	<0.001**^a^**
Agricultural	2115	83.20	428	16.80			498	50.70	484	49.30		
Non-agricultural	1193	88.20	160	11.80			340	59.90	228	40.10		
**Ethnicity**					41.76	<0.001**^a^**					12.64	<0.001**^a^**
Han	3017	86.20	485	13.80			788	55.40	634	44.60		
Other	291	73.90	103	26.10			50	39.10	78	60.90		
**Education level**					11.73	0.008**^a^**					58.81	<0.001**^a^**
No schooling	479	86.90	72	13.10			71	59.70	48	40.30		
Primary school	839	83.60	164	16.40			155	45.20	188	54.80		
Secondary school	1334	83.50	264	16.50			336	48.60	355	51.40		
Higher education	656	88.20	88	11.80			276	69.50	121	30.50		
**Marital status**					4.96	0.084**^a^**					5.09	0.078**^a^**
Unmarried	755	85.90	124	14.10			226	58.90	158	41.10		
Married	2276	84.20	428	15.80			569	52.20	520	47.80		
Other	277	88.50	36	11.50			43	55.80	34	44.20		
**Income**					61.29	<0.001**^a^**					0.08	0.772**^a^**
≤ average	2442	87.70	341	12.30			467	53.70	402	46.30		
> average	866	77.80	247	22.20			371	54.50	310	45.50		
**Employment**					98.43	<0.001**^a^**					21.55	<0.001**^a^**
Employed	1278	78.20	356	21.80			429	48.90	448	51.10		
Unemployed	2030	89.70	232	10.30			409	60.80	264	39.20	0.10	0.661
	***Mean ± SD***		***Mean ± SD***		***Z***	***p***	***Mean ± SD***		***Mean ± SD***		***Z***	***p***
**Time-inconsistent preference**	0.95±0.11		0.92±0.13		-6.188	<0.001**^b^**	0.94±0.11		0.90±0.14		-5.218	<0.001**^b^**
**Self-control**	69.73±8.82		66.58±9.67		-7.27	<0.001**^b^**	69.12±9.26		64.67±9.54		-8.668	<0.001**^b^**
**Negative emotions^b^**	17.78±6.3		16.84±5.97		-3.557	<0.001**^b^**	17.32±6.12		17.06±6.08		-0.754	0.451**^b^**

a Categorical variables: age (categorized), gender, marital status, education level, income, household registration, employment, ethnicity, and alcohol use. Comparisons used the chi-squared test (χ²).

b Continuous variables: time-inconsistent preference, self-control (Tangney Scale), and negative affect (PANAS-NA). The Mann-Whitney U test (MWU) was applied due to non-normal distributions (Shapiro-Wilk p≤0.05).

**Table 4:** Multivariable logistic regression, adjusted associations between time-inconsistent preference and smoking stratified by alcohol use, Sichuan Province, China, 2022

*Variables*	*Non-drinkers*					*Drinkers*		
	*AOR*	*95% CI*		*p*	*AOR*	*95% CI*		*p*
		*Lower*	*Upper*			*Lower*	*Upper*	
**Gender**				<0.001				<0.001
Male ®	1				1			
Female	0.025	0.018	0.035		0.055	0.037	0.080	
**Age** (years)				0.356				0.199
15–44 ®	1				1			
45–59	1.060	0.753	1.493		1.029	0.704	1.504	
≥60	1.289	0.883	1.883		0.745	0.480	1.157	
**Household registration type**				0.036				0.988
Agricultural ®	1				1			
Non-agricultural	0.758	0.586	0.982		1.002	0.758	1.325	
**Ethnicity**				0.003				0.049
Han ®	1				1			
Other	1.680	1.191	2.370		1.601	1.002	2.559	
**Education level**				0.024				<0.001
No schooling ®	1				1			
Primary school	0.927	0.619	1.388		1.22	0.703	2.118	
Secondary school	0.881	0.584	1.328		1.259	0.724	2.191	
Higher education	0.544	0.330	0.897		0.501	0.266	0.945	
**Marital status**				0.057				0.08
Unmarried ®	1				1			
Married	1.528	1.078	2.167		1.389	0.934	2.065	
Other	1.544	0.872	2.732		2.158	1.073	4.340	
**Income**				0.002				0.057
≤ average ®	1				1			
> average	1.516	1.159	1.981		0.757	0.569	1.008	
**Employment**				<0.001				0.003
Employed ®	1				1			
Unemployed	0.442	0.338	0.577		0.634	0.471	0.852	
**Time-inconsistent preference**	0.260	0.102	0.661	0.005	0.253	0.093	0.693	0.008
**Self-control**	0.946	0.933	0.959	<0.001	0.932	0.917	0.946	<0.001
**Negative emotions**	0.969	0.95	0.988	0.002	0.984	0.962	1.007	0.164

AOR: adjusted odds ratio. ® Reference categories.

### Analysis of smoking factors among drinkers and non-drinkers

**Figure 1:**
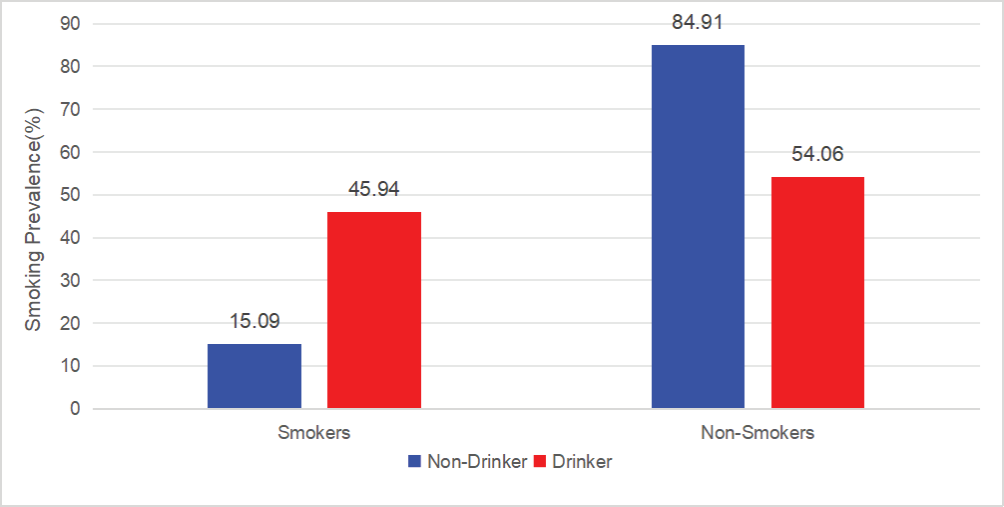
Prevalence of smoking by alcohol use status in Sichuan Province, China, 2022

**Figure 2:**
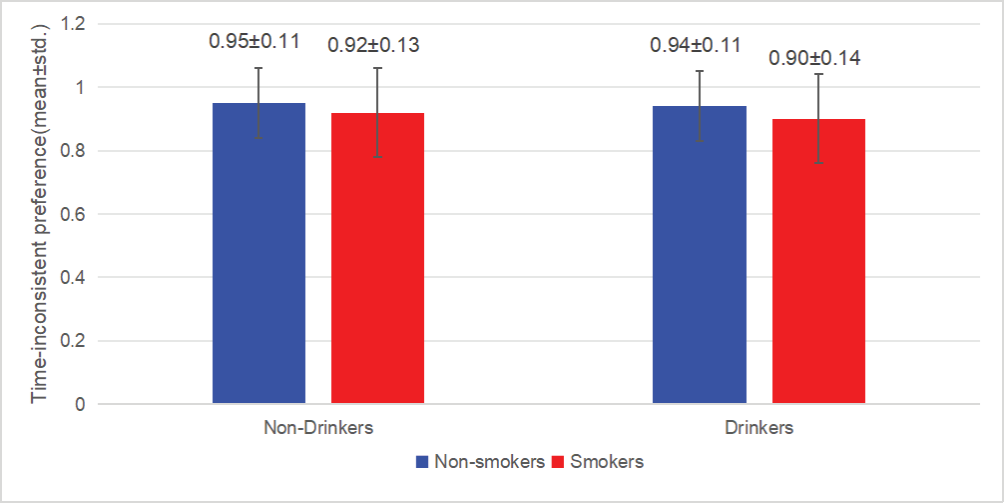
Time-inconsistent preference by smoking and drinking status in Sichuan Province, China, 2022

Analysis of the factors associated with smoking among drinkers and non-drinkers revealed that among non-drinkers, the average time-inconsistent preference of smokers (0.92 ± 0.13) was lower than that of non-smokers (0.95 ± 0.11). There were significant differences between non-smokers and smokers in terms of gender, household registration type, ethnicity, education level, income, employment, time-inconsistent preference, self-control, and negative emotions (all p<0.05). Gender (male reference: AOR=0.025; 95% CI: 0.018–0.035, p<0.001), household registration type (agricultural reference: AOR=0.758; 95% CI: 0.586–0.982, p=0.036), ethnicity (Han reference: AOR=1.68; 95% CI: 1.191–2.37, p=0.003), education level (no school reference: primary school, AOR=0.927; 95% CI: 0.619–1.388; secondary school, AOR=0.881; 95% CI: 0.584–1.328; higher education, AOR=0.544; 95% CI: 0.33–0.897 , p=0.024), income (≤ average reference: AOR=1.516; 95% CI: 1.159–1.981, p=0.002,), employment (employed reference: AOR=1.516; 95% CI: 1.159–1.981, p<0.001), time-inconsistent preference (AOR=0.26; 95% CI: 0.102–0.661, p=0.005), self-control (AOR=0.946; 95% CI: 0.933–0.959, p<0.001), and negative emotions (AOR=0.969; 95% CI: 0.95–0.988, p=0.002) were significantly associated with smoking behavior. Among non-drinkers, those with weaker time-inconsistent preferences (β increase of 0.01 units) were less likely to smoke (AOR=0.26 per 1-unit increase in β, 95% CI: 0.102–0.661, p=0.005). Among the drinkers, the average time-inconsistent preference of smokers (0.90 ± 0.14) was lower than that of non-smokers (0.94 ± 0.11). There were significant differences between non-smokers and smokers in terms of gender, household registration type, ethnicity, education level, income, employment, time-inconsistent preference, self-control, and negative emotions (all p<0.05). Gender (male reference: AOR=0.055; 95% CI: 0.037–0.08, p<0.001), ethnicity (Han reference: AOR=1.601; 95% CI: 1.002–2.559, p=0.049), education level (no schooling reference: primary school, AOR=1.22; 95% CI: 0.703–2.118; secondary school, AOR=1.259; 95% CI: 0.724–2.191; higher education, AOR=0.501; 95% CI: 0.266–0.945 , p<0.001), employment (employed reference: AOR=0.634; 95% CI: 0.471–0.852, p=0.003,), time-inconsistent preference (AOR=0.253; 95% CI: 0.093–0.693, p=0.008), and self-control (AOR=0.932; 95% CI: 0.917–0.946, p<0.001) were significantly associated with smoking behavior. Among drinkers, those with weaker time-inconsistent preferences (β increase of 0.01 units) were less likely to smoke (AOR=0.253 per 1-unit increase in β, 95% CI: 0.093–0.693, p=0.008) (Tables 3 and 4; Figures 1 and 2).

## DISCUSSION

The observed smoking prevalence in this study was slightly lower than the adult smoking rate reported by the Sichuan Provincial Center for Disease Control and Prevention in 2017, lower than the national smoking prevalence among people aged ≥15 years, and similar to rates in recent national surveys^29^. Further, smokers were substantially more prevalent among drinking participants than non-drinking participants. This finding is consistent with the results of other studies. For example, the analysis of a large population cohort, the UK Biobank, in the UK indicated that the prevalence of smoking among drinkers was higher than that of non-drinkers^30^.

This study found that smokers had significantly lower mean time-inconsistent preference values than non-smokers and that time-inconsistent preference was a factor associated with smoking. Moreover, the likelihood of smoking increased with the severity of the time-inconsistent preference. This suggests that smokers’ preferences are time-inconsistent and that their future selves will change the optimal plans made by their previous selves. This finding is consistent with previous studies. For example, the study of Kang and Ikeda^31^ pointed out that the degree of hyperbolic discounting of time-inconsistent preference is positively associated with smoking behavior. In addition, Kossova et al.^32^ measured time-inconsistent preference by personal discount rate and found that a high personal discount rate (i.e. more severe level of time-inconsistent preference) was positively associated with smoking behavior. Finally, through a comprehensive analysis of 69 studies on the impact of time-inconsistent preference on smoking, Barlow et al.^33^ found that 44 of the studies concluded that smokers were better able to discount for the future than non-smokers and that higher discounting was associated with smoking. These studies together reveal the close connection between time-inconsistent preference and smoking behavior.

Further analysis of factors associated with smoking behavior among drinking and non-drinking survey participants revealed that time-inconsistent preference is a factor influencing smoking among both drinkers and non-drinkers and that the likelihood of smoking increases with the severity of the time-inconsistent preference. This finding shows that time-inconsistent preference shows a direct and robust association with smoking behavior. Although drinking behavior is associated with smoking behavior, it does not significantly modify the association of time-inconsistent preference on smoking behavior, indicating that drinking is not a confounding factor in this relationship. Time-inconsistent preference can be regarded as an important and stable associated factor of smoking behavior. Time-inconsistent preference stems from the projection bias and hindsight bias that smokers have when making intertemporal choices. Scholars have shown that participants with high time perception levels can regulate their time-inconsistent preferences when making intertemporal choices and are willing to sacrifice instant gratification for delayed options with greater benefits, while participants with low time perception levels are more inclined to choose options with small but immediate benefits^34^. Therefore, time perception represents a testable target for behavioral interventions. Deposit contracts exemplify a commitment mechanism that merits controlled evaluation. Previous studies have confirmed that some behavioral economics interventions, such as the financial commitment smoking cessation interventions based on deposit contracts, may potentially improve time perception and support smoking reduction^35,36^. The subjects of such interventions bet money on their successful smoking cessation and earn back their pre-deposited funds by achieving a behavioral goal – quitting smoking. This approach aims to reduce impulsive choices by pre-committing to long-term goals. This could potentially strengthen resistance to immediate temptations, prevent impulsive decision-making, bolster the ability to withstand such temptations, and ensure the protection of long-term interests.

### Limitations

Despite its contributions, this study has limitations. First, the cross-sectional design precludes causal inferences regarding the time-inconsistent preferences-smoking relationship. Longitudinal or experimental studies are needed to establish temporality and causality. Second, generalizability may be limited as data were collected solely in Sichuan Province. Cultural, socioeconomic, and policy differences may restrict applicability to other regions or countries. Third, although key sociodemographic and psychological confounders (e.g. alcohol use, self-control, negative emotion) were adjusted for, residual confounding from unmeasured factors (e.g. genetic predisposition, peer influence, environmental triggers) cannot be excluded. Fourth, we focused primarily on the direct association between time-inconsistent preferences and smoking without comprehensively exploring potential mediating mechanisms (e.g. neurocognitive or environmental pathways). Finally, tobacco use was analyzed as a binary behavior (smoker, non-smoker) without further categorization (e.g. by frequency, dependence severity, cessation attempts), potentially obscuring nuanced relationships between time-inconsistent preferences and smoking subtypes. Future studies should investigate mediating pathways and behavioral stratifications.

## CONCLUSIONS

This study found that approximately one-fourth of surveyed individuals aged ≥15 years, in Sichuan Province, were smokers and the mean time-inconsistent preference value of smokers was significantly lower than that of non-smokers; the time-inconsistent preference showed a significant negative association with smoking behavior. In addition, time-inconsistent preference was a factor associated with smoking behavior in both drinkers and non-drinkers, with the likelihood of smoking increasing with the severity of the preference. Given the cross-sectional nature of this study, which identifies associations but cannot establish causality, these findings highlight the need for further research. Future longitudinal or cohort studies are warranted to confirm the temporal relationship between time-inconsistent preferences and smoking initiation or cessation, and to explore the underlying mechanisms. Research exploring interventions incorporating insights from behavioral economics, such as commitment devices or incentives, could be valuable to assess their potential effectiveness in supporting smoking cessation efforts within this context. Further investigation is needed to determine if such approaches could strengthen expectations of long-term health benefits and aid behavior change.

## Supplementary Material



## Data Availability

The data supporting this research are available from the authors on reasonable request.
