# Supplementary material for: Exploring the association of time-inconsistent preferences with smoking behavior: A cross-sectional survey study from Sichuan, China
Source: Tob Induc Dis. 2025 Sep 24;23:10.18332/tid/209192. doi: 10.18332/tid/209192 (PMC12459343; doi:10.18332/tid/209192)
Supplement: Supplementary file 1 [file TID-23-139-s1.pdf]

Supplementary Measurement of time-inconsistent preference

In the following questions, you will face two different scenarios in which you must make choices according to your actual inclinations. After you have made your choices, we will randomly select a question and decide, by way of rolling dice, whether you get the corresponding amount. We will pay real cash according to the time and amount of the selected question. In this scenario you will choose between receiving a smaller sum of money sooner or a larger sum of money later. There are two scenario events: “Get 100 yuan tomorrow" vs. "Get more money in one month" and “Get 100 yuan in 12 months" vs. “Get more money in 13 months." Now please make your choices:

1. A. Get ￥100 tomorrow	B. Get ￥110 after one month
2. A. Get ￥100 tomorrow	B. Get ￥120 after one month
3. A. Get ￥100 tomorrow	B. Get ￥130 after one month
....	
8. A. Get ￥100 after 12 months	B. Get ￥110 after 13 months
9. A. Get ￥100 after 12 months	B. Get ￥120 after 13 months
10. A. Get ￥100 after 12 months	B. Get ￥130 after 13 months

© 2025 Li Y. et al.
